# IGSF10 regulates osteogenesis and osteoclastogenesis via a noncanonical EGFR–STAT1 signaling axis to promote skeletal regeneration

**DOI:** 10.1038/s41413-026-00552-2

**Published:** 2026-08-04

**Authors:** Jin Wen, Yuwei Deng, Ruixue Jiang, Longwei Hu, Qianju Wu, Xiao Wang, Zheting Liao, Wenjing Ding, Fei Jiang, Xi Chen, Xiaohan Ma, Chunhua Yu, Xinquan Jiang

**Affiliations:** 1 Department of Prosthodontics, Shanghai Ninth People’s Hospital, Shanghai Jiao Tong University School of Medicine, Shanghai, PR China; 2 College of Stomatology, Shanghai Jiao Tong University, Shanghai, PR China; 3 National Center for Stomatology, Shanghai, PR China; 4 National Clinical Research Center for Oral Diseases, Shanghai, PR China; 5 Shanghai Key Laboratory of Stomatology, Shanghai, PR China; 6 Shanghai Research Institute of Stomatology, Shanghai, PR China; 7 Shanghai Engineering Research Center of Advanced Dental Technology and Materials, Shanghai, PR China; 8 Department of Oral & Maxillofacial-Head & Neck Oncology, Shanghai Ninth People’s Hospital, Shanghai Jiao Tong University School of Medicine, Shanghai, PR China; 9 Stomatological Hospital of Xiamen Medical College, Xiamen, PR China; 10 Xiamen Key Laboratory of Stomatological Disease Diagnosis and Treatment, Xiamen, PR China; 11 Joint Surgery Department, the First Affiliated Hospital of Guangzhou Medical University, Guangzhou, PR China; 12 Library, Shanghai Jiao Tong University School of Medicine, Shanghai, PR China; 13 Department of General Dentistry Affiliated Hospital of Stomatology, Nanjing Medical University, Nanjing, PR China; 14 Department of Preventive Dentistry, Shanghai Ninth People’s Hospital, Shanghai Jiao Tong University School of Medicine, Shanghai, PR China; 15 Division of Biomaterials and Tissue Engineering, University College London (UCL) Eastman Dental Institute, Royal Free Hospital, UCL Medical School, Rowland Hill Street, London, NW3 2PF UK; 16 Shanghai Stomatological Hospital Fudan University, Shanghai, PR China

**Keywords:** Bone, Homeostasis

## Abstract

Bone remodeling requires precise coordination between osteoblast-mediated bone formation and osteoclast-driven resorption. However, directly targetable and therapeutically actionable mediators that can coordinately modulate both processes during regeneration remain relatively limited. Here, we identify immunoglobulin superfamily member 10 (IGSF10) as a dual-function modulator of bone remodeling. *Igsf10-*deficient mice exhibit reduced bone mass, elevated osteoclast activity, and impaired osteogenesis. Recombinant IGSF10 protein restores osteogenic capacity and suppresses osteoclastogenesis in knockout cells, while exerting pro-osteogenic and anti-resorptive effects in wild-type mesenchymal and monocyte-derived cultures. Mechanistically, IGSF10 activates a noncanonical EGFR–STAT1 signaling pathway, distinct from BMP2–Smad signaling. Co-immunoprecipitation and molecular docking confirm IGSF10–EGFR interaction, and blockade of EGFR abrogates IGSF10-induced osteogenesis and its inhibitory effects on osteoclastogenesis. In vivo, IGSF10 promotes bone regeneration in both calvarial and periodontal defect models and exhibits synergy with subtherapeutic BMP2. These findings position IGSF10 as a previously unrecognized dual-acting regulator that coordinates bone formation and resorption in a context-dependent manner, with potential therapeutic value for craniofacial and skeletal regeneration.

## Introduction

Bone regeneration is essential for restoring skeletal function following trauma, infection, tumor resection, or congenital defects.^1^ Although bone exhibits a remarkable intrinsic capacity for repair, large or critical-sized defects often fail to heal spontaneously, necessitating clinical intervention.^2^ Addressing these non-healing injuries remains a clinical priority in orthopedics, dentistry, and craniofacial reconstruction.

Physiological bone remodeling is governed by a dynamic balance between bone formation by osteoblasts and bone resorption by osteoclasts.^3,4^ This tightly coupled process ensures skeletal adaptation to mechanical loading,^5^ maintenance of mineral homeostasis, and repair of microdamage. Disruption of this balance contributes to metabolic bone diseases such as osteoporosis, but also to impaired fracture healing and compromised bone quality.^3^ Notably, many regenerative approaches emphasize enhancing osteoblast-driven bone formation; however, insufficient consideration of osteoclast-mediated resorption can impair the remodeling and shaping of newly formed bone, ultimately compromising the quality and functionality of regenerated tissue.^6^

A prime example is bone morphogenetic protein 2 (BMP2), a potent osteoinductive factor approved for clinical use in selected orthopedic and dental indications.^7^ BMP2 promotes robust osteoblast differentiation,^8^ but it does not directly or selectively suppress osteoclast differentiation or activity.^9^ Consequently, supraphysiological doses are often required to achieve therapeutic efficacy, which can lead to adverse effects such as ectopic ossification, excessive inflammatory reactions, and irregular bone architecture^10–12^—limitations that are particularly problematic in oral and craniofacial applications. These clinical challenges have motivated ongoing efforts to identify alternative osteogenic factors and/or combination strategies that maintain efficacy while enabling BMP2 dose reduction.

These considerations highlight the need for next-generation therapeutic strategies that achieve a more balanced modulation of bone remodeling—enhancing osteogenesis while appropriately constraining resorption in a context-dependent manner. Multiple signaling pathways—including the RANKL/OPG axis, sclerostin-mediated Wnt regulation, growth factors such as transforming growth factor-β (TGF-β) and insulin-like growth factor-1 (IGF-1), as well as developmental regulators including Wnt-associated cues (e.g., Sema3A) and Ephrin family members—are known to influence both osteoblast and osteoclast programs.^13–16^ While these factors are indispensable for skeletal development and homeostasis, many function as broadly acting morphogenetic or growth signals with pleiotropic effects, complex feedback regulation, and potential translational constraints when applied therapeutically.^17^ As a result, therapeutically actionable mediators that can be readily harnessed to coordinately and context-dependently regulate osteoblast and osteoclast programs during bone regeneration—while offering a favorable therapeutic window—remain comparatively limited.

Members of the immunoglobulin superfamily (IgSF) have emerged as important regulators of cell–cell communication, immune regulation, and tissue patterning.^18^ Within the skeletal system, specific IgSF members have been implicated in bone remodeling; for example, IGSF11 facilitates osteoclast precursor fusion and contributes to pathological bone loss, with effects largely restricted to the resorptive arm of remodeling.^19–21^ Whether and how other IgSF proteins coordinate osteoblast and osteoclast activities during bone regeneration remains poorly understood.

Immunoglobulin superfamily member 10 (IGSF10) is a large secreted glycoprotein containing multiple immunoglobulin-like (Ig-like) and leucine-rich repeat domains.^22^ Initially characterized for its role in hypothalamic development,^23,24^ IGSF10 has also been associated with cancer suppression and epithelial morphogenesis.^25–27^ More recent evidence points to a potential skeletal function for IGSF10. Transcriptomic profiling reveals *Igsf10* upregulation in mechanically stimulated calvarial osteogenic cells, while in situ hybridization confirms its elevated expression specifically within the healing fracture callus, suggesting a potential role in load-responsive remodeling.^22^ In parallel, a frameshift mutation in IGSF10 was identified in a family with cleidocranial dysplasia (CCD) lacking RUNX2 mutations. The mutation co-segregated with craniofacial and clavicular abnormalities, implicating IGSF10 in skeletal development.^28^ Together, these transcriptional and clinical observations point toward a potential role for IGSF10 in bone biology.

Despite these suggestive findings, IGSF10 has not been functionally characterized in bone, and its roles in regulating osteoblast and osteoclast activity, as well as the underlying molecular mechanisms, remain unknown. In particular, whether IGSF10 can act as a context-dependent modulator of bone remodeling with dual effects on formation and resorption has not been explored.

Here, we investigated the role of IGSF10 as a putative dual-function regulator of bone remodeling and regeneration. Using *Igsf10*-deficient mouse models, we examined the impact of gene deletion on bone homeostasis across multiple anatomical sites. We further assessed the effects of exogenous IGSF10 on osteogenesis, osteoclastogenesis, and extended the analysis to in vivo bone regeneration using calvarial and periodontal defect models, and further investigated its combinatorial effects with BMP2. Notably, we evaluated the therapeutic potential of IGSF10 in human periodontal ligament cells (PDLCs), a clinically relevant but underexplored cell population in craniofacial bone repair. Mechanistically, we investigated the downstream signaling mechanisms, focusing on the epidermal growth factor receptor (EGFR)–signal transducer and activator of transcription 1 (STAT1) axis. This was motivated by the clinical need for BMP2 dose-sparing strategies and by the mechanistic premise that an EGFR–STAT1–biased pathway may complement canonical BMP/Smad signaling. Collectively, this study aims to define the functional role, mechanistic basis, and therapeutic promise of IGSF10 in bone remodeling and skeletal regeneration.

## Results

### Systemic skeletal phenotype of *Igsf10*-deficient mice

**Fig. 1:**
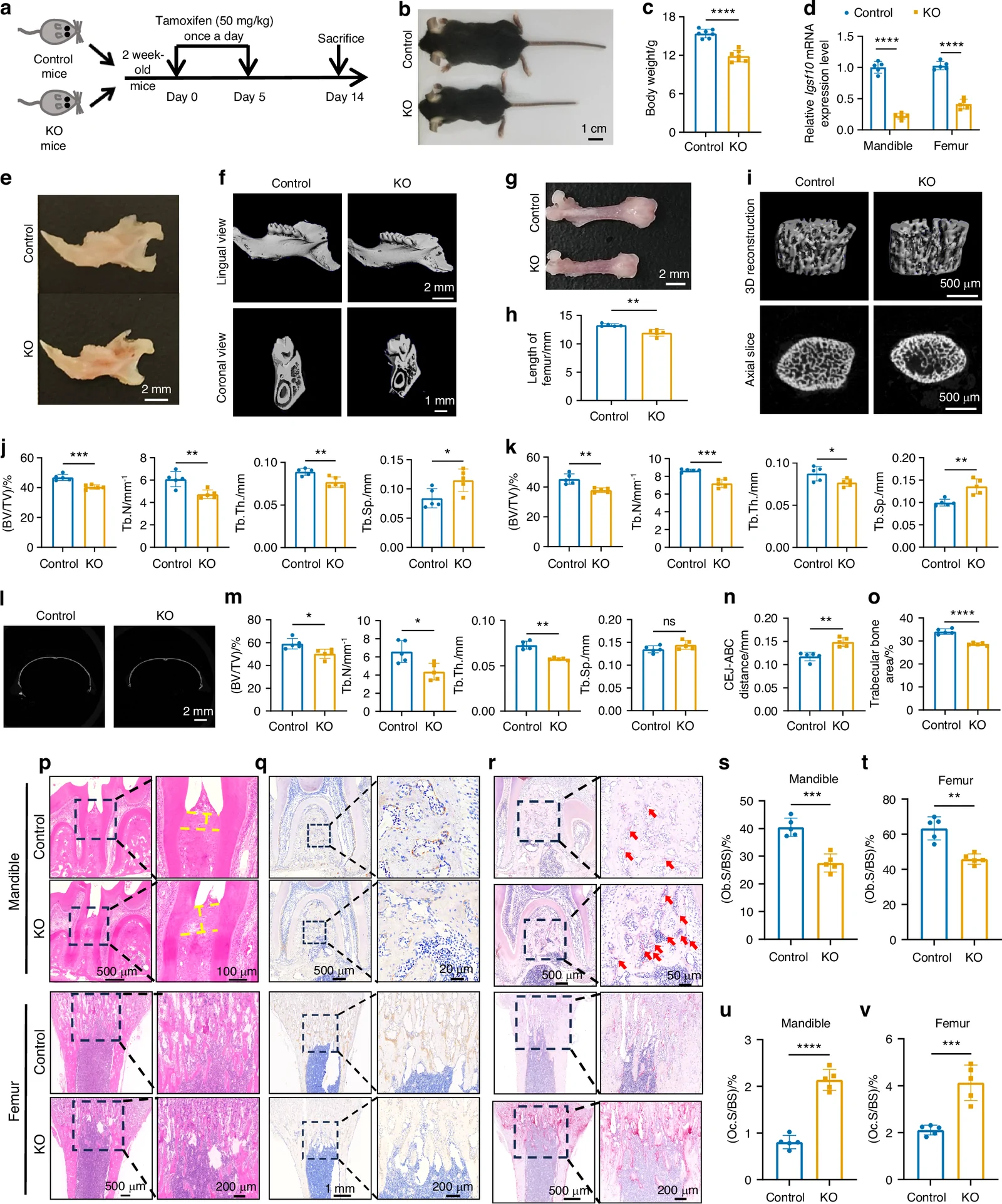
*Igsf10* deficiency impairs postnatal skeletal development and remodeling. **a** Experimental timeline for tamoxifen-induced *Igsf10* knockout. **b** Representative images of 4-week-old control and KO mice. **c** Body weight (*n* = 7). **d** *Igsf10* gene expression level in mandible and femur by qRT-PCR (*n* = 5). **e** Mandible morphology. **f** 3D micro-CT reconstructions of mandible. Femur morphology (**g**) and length quantification (**h**) (*n* = 5). **i** 3D micro-CT reconstruction of femoral trabecular bone. **j** Micro-CT quantification of trabecular parameters in mandible: BV/TV, Tb.N, Tb.Th, Tb.Sp (*n* = 5). **k** Micro-CT quantification of trabecular parameters in femur: BV/TV, Tb.N, Tb.Th, Tb.Sp (*n* = 5). **l** Representative image of the 3D micro-CT reconstruction of the calvaria. **m** Micro-CT quantification of trabecular parameters in calvaria: BV/TV, Tb.N, Tb.Th, Tb.Sp (*n* = 5). **n** Quantification of CEJ–ABC distance (*n* = 5). **o** Quantification of the percentage of trabecular bone area (*n* = 5). **p** H&E staining in mandible (yellow dashed line: CEJ–ABC distance) and femur. **q** Ocn IHC staining in mandible and femur. **r** TRAP staining in mandible and femur (red arrow: TRAP-positive multinucleated osteoclast). Quantification of Ob.S/BS (**s**–**t**) and Oc.S/BS (**u**–**v**) in mandible and femur (*n* = 5)

To assess the role of *Igsf10* in postnatal skeletal development, we generated tamoxifen-inducible knockout (KO) mice and evaluated their phenotype at 4 weeks of age. Tamoxifen (50 mg/kg) was administered intraperitoneally once daily from postnatal day 14 for five consecutive days, and tissues were harvested at week 4 (Fig. 1a). KO mice exhibited a smaller body size and a significant reduction in body weight (11.87 ± 0.88 g) compared with control mice (15.40 ± 0.67 g; *P* < 0.000 1; Fig. 1b, c). Quantitative reverse transcription PCR (qRT-PCR) confirmed efficient *Igsf10* deletion in both mandibles and femurs (Fig. 1d). Mandibles were visibly smaller (Fig. 1e), and femur length was significantly shortened in KO mice compared to controls (11.94 ± 0.58 mm vs. 13.30 ± 0.26 mm; *P* < 0.005; Fig. 1g, h).

Three-dimensional micro-CT reconstructions revealed markedly reduced bone mass in mandibles, femurs, and calvaria of KO mice (Fig. 1f, i, l). Trabecular micro-CT quantification further confirmed significant trabecular bone deterioration in mandibles (Fig. 1j), bone volume fraction (BV/TV) was reduced from 46.85% (Control) to 40.46% (KO), trabecular thickness (Tb.Th) decreased by 0.012 mm, trabecular number (Tb.N) declined by 1.345 mm⁻¹, and trabecular separation (Tb.Sp) increased by 0.031 mm. Consistent changes were observed in femurs (Fig. 1k), where BV/TV dropped from 45.35% to 37.86%, Tb.Th was reduced by 0.011 mm, Tb.N decreased by 1.462 mm⁻¹, and Tb.Sp increased by 0.036 mm. The calvaria showed a distinct pattern, with BV/TV decreasing from 59.08% to 50.22%, Tb.Th reducing by 0.015 mm, and Tb.N declining by 2.214 mm⁻¹, whereas Tb.Sp showed no significant alteration (Control: 0.135 ± 0.008 mm vs. KO: 0.144 ± 0.009 mm, *P* > 0.05) (Fig. 1m). Cortical analysis of femurs at 4 weeks indicated a significant reduction in cortical area (Ct.Ar, from 8.038 ± 0.493 mm² to 6.966 ± 0.272 mm²) with the absence of a significant change in cortical thickness (Ct.Th, 0.128 ± 0.002 mm vs. 0.123 ± 0.005 mm, *P* = 0.096) (Fig. S1).

To determine whether the trabecular phenotype persists beyond early postnatal development, we analyzed an independent cohort at 12 weeks of age; trabecular deficits remained evident in femurs at this mature stage (Fig. S2).

### Loss of osteogenic capacity and increased osteoclast activity in *Igsf10*-deficient mice

Histological analyses corroborated the skeletal defects observed by micro-CT. Hematoxylin and eosin (H&E) staining of mandibles revealed an increased cementoenamel junction–alveolar bone crest (CEJ–ABC) distance in KO mice (Control: 0.117 ± 0.009 mm vs. KO: 0.149 ± 0.010 mm), consistent with mandibular bone loss (Fig. 1n, p). In femurs, KO samples showed a 16.07% reduction in trabecular bone area compared with controls (Fig. 1o, p).

Osteocalcin (Ocn) immunohistochemistry followed by histomorphometric quantification demonstrated reduced osteoblast surface per bone surface (Ob.S/BS, %) in both mandibles and femurs of KO mice: specifically, in mandibles, Ob.S/BS was significantly decreased from 40.48% ± 3.303% to 27.56% ± 3.235%, while in femurs, Ob.S/BS was reduced from 63.32% ± 6.570% to 45.89% ± 2.904% (Fig. 1q, s, t). In contrast, tartrate-resistant acid phosphatase (TRAP) staining revealed increased numbers of TRAP-positive multinucleated osteoclasts in both sites, with the osteoclast surface per bone surface (Oc.S/BS) elevated by 2.65‑fold in mandibles and by 1.95‑fold in femurs (Fig. 1r, u, v), indicating enhanced osteoclastogenesis and resorptive activity in vivo.

### IGSF10 rescues impaired osteogenesis and suppresses osteoclastogenesis in *Igsf10*-deficient cells

**Fig. 2:**
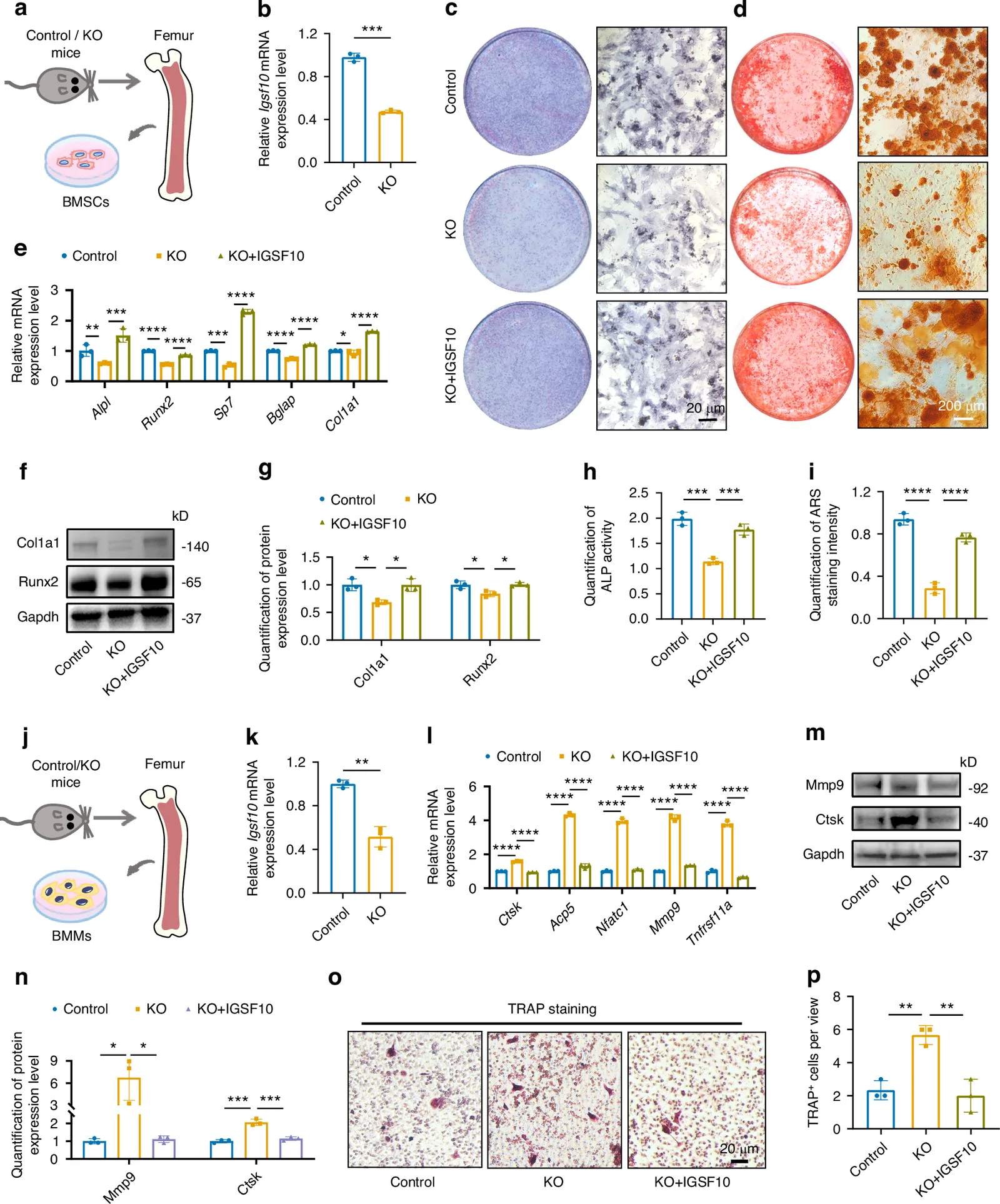
IGSF10 rescues osteogenic defects and suppresses osteoclastogenesis in *Igsf10*-deficient cells. **a** Schematic diagram of BMSCs isolation. **b** qRT-PCR validation of *Igsf10* knockout in BMSCs (*n* = 3). **c** ALP staining (day 7). **d** ARS staining (day 14). **e** Osteogenic gene expression (*Alpl*, *Runx2*, *Sp7*, *Bglap*, *Col1a1*) in BMSCs on day 3 (*n* = 3). **f** Western blot of Col1a1 and Runx2 in BMSCs (day 7). **g** Quantification of protein expression levels in (**f**) (*n* = 3). **h** Quantification of ALP activity (*n* = 3). **i** Quantification of ARS staining (*n* = 3). **j** Schematic diagram of BMMs isolation. **k** qRT-PCR validation of *Igsf10* knockout in BMMs (*n* = 3). **l** Osteoclastogenic gene expression (*Ctsk, Acp5, Nfatc1, Mmp9, Tnfrsf11a*) in BMMs on day 7 (*n* = 3). **m** Western blot of Mmp9 and Ctsk in BMMs (day 7). **n** Quantification of protein expression levels in (**m**) *(n* = 3). **o** TRAP staining of BMMs-derived osteoclasts. **p** Quantification of TRAP-positive multinucleated cells (*n* = 3)

To explore cellular mechanisms, we evaluated lineage commitment of bone marrow-derived progenitors in vitro using bone marrow-derived mesenchymal stromal cells (BMSCs) and bone marrow-derived monocytes (BMMs) (Fig. 2a, j). qRT-PCR confirmed efficient deletion of *Igsf10* in both BMSCs and BMMs isolated from KO mice (Fig. 2b, k).

Under osteogenic induction, KO-derived BMSCs displayed reduced mRNA levels of osteogenic markers (*Alpl*, *Runx2*, *Sp7*, *Bglap*, and *Col1a1*) at day 3 (Fig. 2e). Consistently, Col1a1 and Runx2 protein levels were decreased at day 7 (Fig. 2f, g). Functional assays revealed a 42.56% decrease in ALP activity at day 7 (Fig. 2c, h) and a 69.46% reduction in mineral deposition by ARS staining at day 14 (Fig. 2d, i). Recombinant IGSF10 largely restored osteogenic marker expression and mineralization in KO BMSCs (Fig. 2c–i).

In parallel, BMMs were induced toward osteoclasts with RANKL and M-CSF. KO-derived cultures exhibited increased expression of osteoclast-associated genes (*Ctsk*, *Acp5*, *Nfatc1*, *Mmp9*, and *Tnfrsf11a*) (Fig. 2l), accompanied by elevated Mmp9 and Ctsk protein levels (Fig. 2m, n). The number of TRAP‑positive multinucleated osteoclasts was nearly 2-fold higher in KO cultures than in controls, confirming the functional impact of the altered molecular landscape (Fig. 2o, p). Addition of recombinant IGSF10 attenuated osteoclast differentiation in KO BMM cultures, reducing both TRAP-positive cell numbers and osteoclastic marker expression toward control levels (Fig. 2l–p).

### Exogenous IGSF10 promotes osteogenesis and suppresses osteoclastogenesis in BMSCs and THP-1 cells

**Fig. 3:**
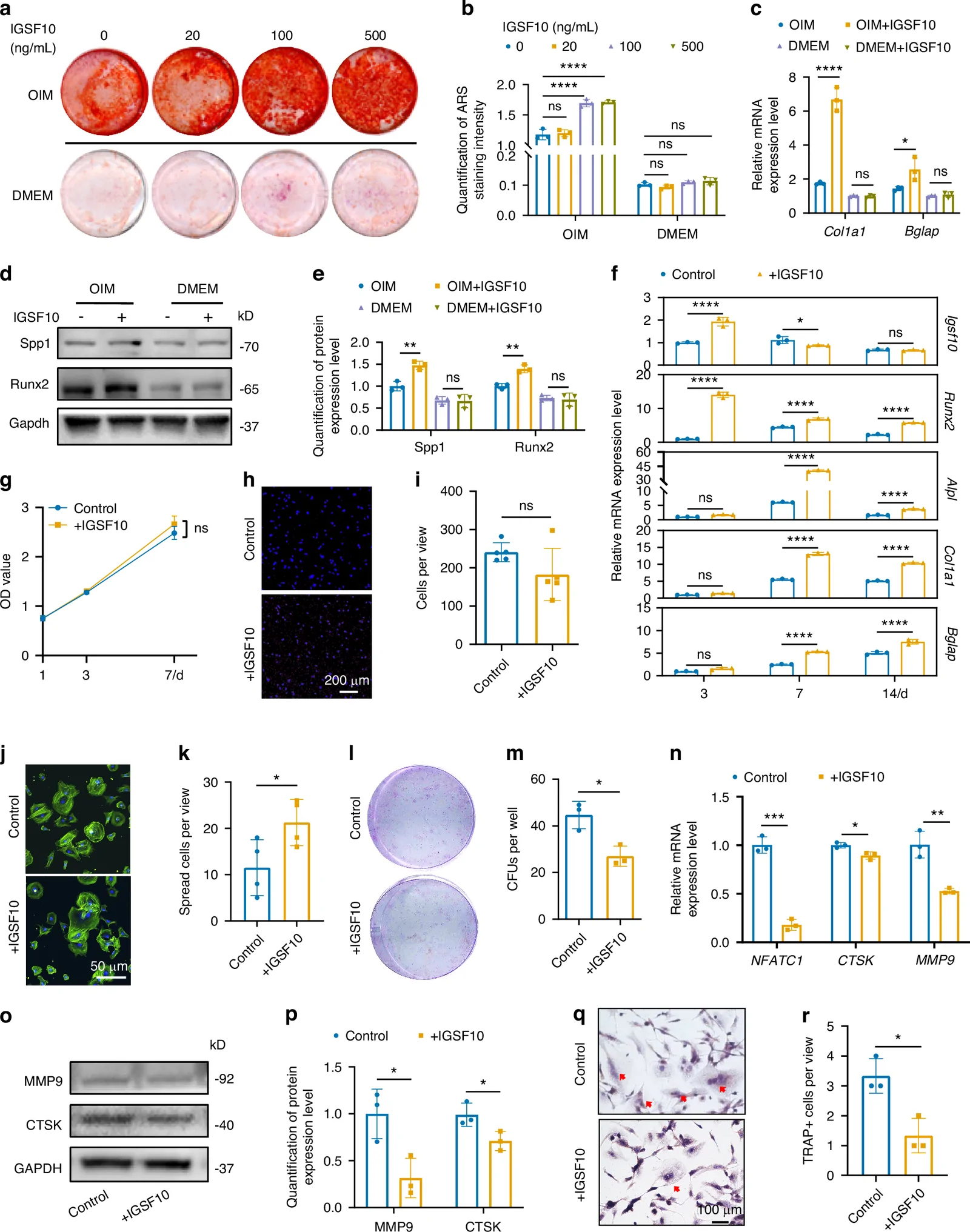
Pro-osteogenic and anti-osteoclastic effects of exogenous IGSF10 protein in vitro. **a** ARS staining of BMSCs treated with IGSF10 under OIM or DMEM (day 14). **b** Quantification of ARS staining (*n* = 3). **c** *Col1a1* and *Bglap* mRNA in BMSCs (day 3) (*n* = 3). **d** Western blot of Spp1, Runx2 in BMSCs (day 7). **e** Quantification of protein expression levels in (**d**) (*n* = 3). **f** qRT-PCR detection of *Igsf10*, *Runx2*, *Alpl*, *Col1a1*, and *Bglap* gene expression in BMSCs after 3, 7, and 14 days of induction (*n* = 3). **g** BMSCs proliferation by CCK-8 assay (*n* = 5). **h** Cell adhesion at 2 h. **i** Quantification of adherent cells per view in (**h**) (*n* = 5). **j** Representative images of BMSCs morphological spreading. **k** Quantification of fully spread cells in (**j**) (*n* = 4). **l** CFU assay of BMSCs. **m** CFU count (*n* = 3). **n** Osteoclastogenic gene (*NFATC1*, *CTSK*, *MMP9*) expression in THP-1 cells (*n* = 3). **o** Western blot of MMP9 and CTSK in THP-1 cells. **p** Quantification of protein expression levels in (**o**) (*n* = 3). **q** TRAP staining of THP-1-derived osteoclasts (red arrow: TRAP-positive multinucleated cell). **r** Quantification of TRAP-positive multinucleated cells (*n* = 3)

To determine whether IGSF10 directly regulates osteogenic and osteoclastic programs, we treated wild-type BMSCs and THP-1-derived osteoclast precursors with recombinant IGSF10 in vitro. In BMSCs cultured in osteogenic induction medium (OIM), IGSF10 enhanced mineral deposition in a dose-dependent manner as assessed by ARS staining at day 14. Notably, the 100 ng/mL IGSF10 treatment group showed a 44.37% increase in ARS staining intensity compared to the control group, with corresponding quantification provided in Fig. 3a, b. In contrast, no increase in mineralization was observed under standard growth medium (DMEM), indicating that IGSF10 potentiates osteogenesis in a permissive induction context. Based on ARS quantification, 100 ng/mL IGSF10 was used for subsequent experiments. At this dose, IGSF10 increased *Col1a1* and *Bglap* mRNA expression at day 3 (Fig. 3c) and elevated Spp1 and Runx2 protein levels at day 7 (Fig. 3d, e). A time-course analysis further showed that exogenous IGSF10 promoted earlier and/or higher expression of both early (*Runx2*, *Alpl*, *Col1a1*) and late (*Bglap*) osteogenic markers during induction (Fig. 3f).

Cell Counting Kit-8 (CCK-8) assays showed no detectable effect on BMSCs viability/proliferation (Fig. 3g), and Annexin V/PI staining revealed no increase in apoptosis (Fig. S3). Cell adhesion at 2 h was comparable between groups (241 ± 25 vs. 182 ± 68 cells/view; Fig. 3h, i), whereas at 4 h IGSF10-treated BMSCs exhibited increased spreading and cytoskeletal organization (Fig. 3j, k). CFU assays showed a modest reduction in clonogenicity (45 ± 6 vs. 27 ± 4 CFUs/well; Fig. 3l, m), consistent with a shift toward differentiation under osteogenic cues.

In THP-1-derived osteoclast cultures induced by RANKL and M-CSF, IGSF10 reduced osteoclast-associated transcripts and protein levels (MMP9, CTSK, NFATc1) (Fig. 3n–p). Consistent with these molecular changes, IGSF10 reduced the formation of TRAP‑positive multinucleated osteoclasts by approximately 60% (Fig. 3q, r).

### IGSF10 enhances osteogenic differentiation in PDLCs in vitro and promotes periodontal bone regeneration in vivo

**Fig. 4:**
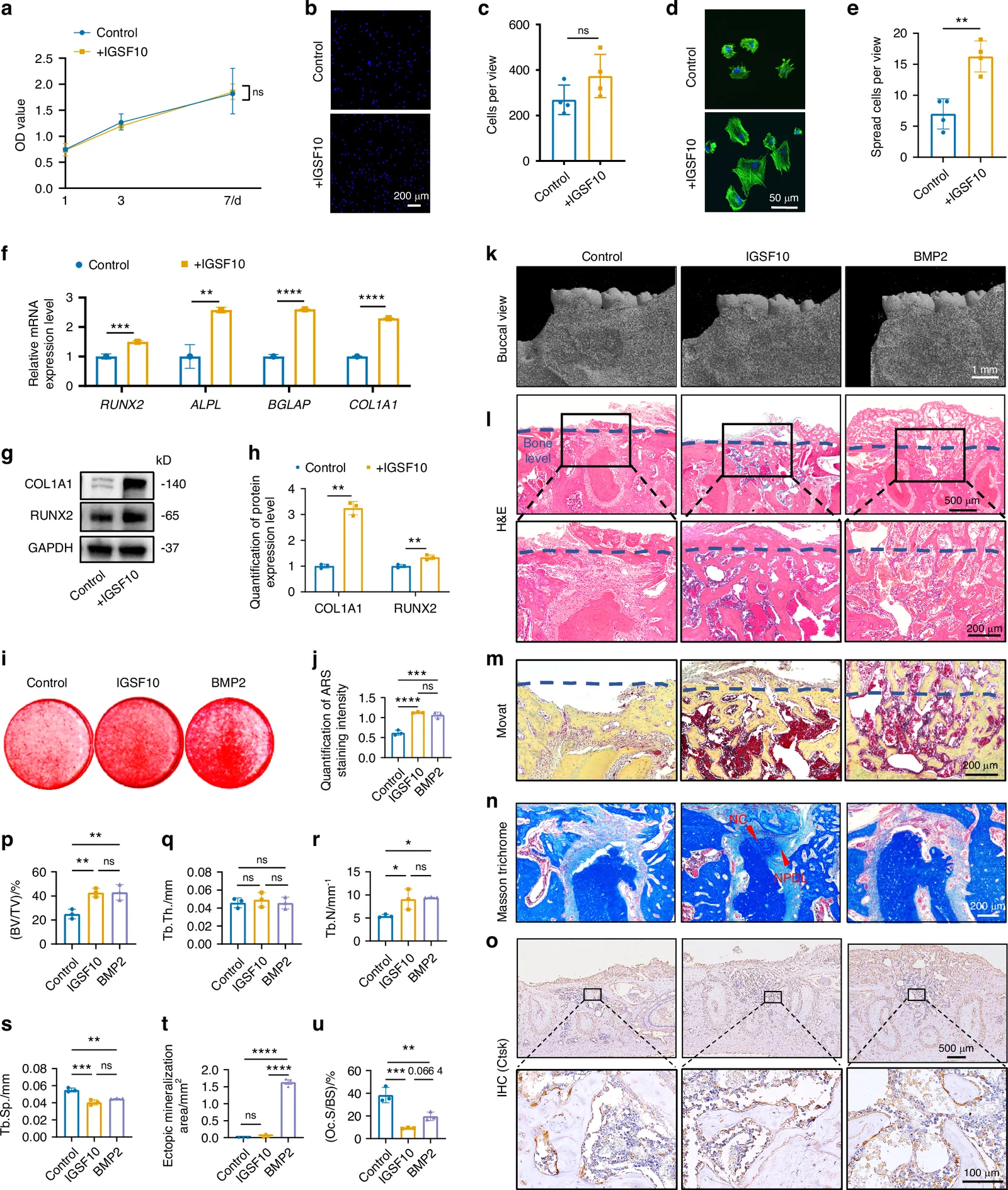
IGSF10 enhances PDLCs osteogenesis and periodontal regeneration. **a** PDLCs proliferation by CCK-8 assay (*n* = 5). **b** PDLCs adhesion at 2 h. **c** Quantification of adherent cells per view in (**b**) (*n* = 4). **d** Representative images of PDLCs morphological spreading. **e** Quantification of fully spread cells in (**d**) (*n* = 4). **f** Osteogenic gene mRNA (*RUNX2*, *ALPL*, *BGLAP*, *COL1A1*) in PDLCs (day 3) by qRT-PCR (*n* = 3). **g** Western blot of COL1A1 and RUNX2 in PDLCs (day 7). **h** Quantification of protein expression levels in (**g**) (*n* = 3). **i** ARS staining of PDLCs (day 14). **j** Quantification of ARS staining (*n* = 3). **k** 3D micro-CT reconstruction of rat mandibular defects (day 21). **l** H&E staining of the defect area at low and high magnification (blue dashed line: the boundary of the native alveolar bone). **m** Movat staining of the defect area. **n** Masson trichrome staining of the defect area. NC new cementum, NPDL new periodontal ligament. **o** Ctsk immunohistochemical staining of the defect area. Quantitative micro-CT analysis of regenerated bone: BV/TV (**p**), Tb.Th (**q**), Tb.N (**r**), Tb.Sp (**s**) (*n* = 3). **t** Quantification of ectopic mineralization area in H&E staining (the area of newly formed bone extending beyond the blue dashed line) (*n* = 3). **u** Quantification of Oc.S/BS in Ctsk staining (*n* = 3)

We next evaluated IGSF10 in human PDLCs. IGSF10 did not affect PDLCs’ viability/proliferation (Fig. 4a). Initial attachment at 2 h was unchanged (Fig. 4b, c), whereas at 4 h IGSF10-treated PDLCs showed increased spreading and cytoskeletal extensions (Fig. 4d, e).

Under osteogenic induction, IGSF10 increased the expression of osteogenic regulators (*RUNX2*, *ALPL*, *BGLAP*, and *COL1A1*) at the mRNA level (Fig. 4f) and elevated COL1A1 and RUNX2 protein levels (Fig. 4g, h). ARS staining at day 14 showed robust mineralization comparable to BMP2 (Fig. 4i, j). Quantitative analysis of the cell lysates further indicated that the calcium content reached 2.337 ± 0.004 µg/mL in the IGSF10 group, showing no statistically significant difference from the BMP2 group (2.367 ± 0.014 µg/mL, *P* > 0.05; Fig. S4).

For in vivo validation, we used a rat periodontal defect model with collagen gel scaffolds loaded with IGSF10 (1 500 ng/mL) or BMP2 as a positive control. Micro-CT and histological analyses at day 21 showed robust defect repair in the IGSF10 group with improved trabecular organization and integration (Fig. 4k–n). Quantitative micro-CT analysis demonstrated that IGSF10 treatment significantly increased BV/TV (42.74% ± 3.605%) and Tb.N (9.048 ± 2.216 mm⁻¹) while decreasing Tb.Sp (0.040 ± 0.003 mm) compared to the control group. Notably, these parameters in the IGSF10 group were comparable to those in the BMP2-treated group (BV/TV: 43.03% ± 6.396%; Tb.N: 9.427 ± 0.063 mm⁻¹; Tb.Sp: 0.045 ± 0.001 mm; *P* > 0.05), with no significant difference observed in Tb.Th among all groups (Fig. 4p–s). Ectopic mineralization area beyond the defect boundary was lower in the IGSF10 group (0.042 ± 0.054 mm^2^) than in the BMP2 group (1.636 ± 0.119 mm^2^; *P* < 0.000 1) (Fig. 4l, t). Ctsk immunohistochemistry and Oc.S/BS quantification indicated reduced osteoclast activity in IGSF10-treated defects, showing a 75.69% reduction versus the control group and a trend toward reduction versus the BMP2 group (52.71% lower; *P* = 0.066 4) (Fig. 4o, u).

### IGSF10 promotes osteogenesis via STAT1 activation independently of BMP2

**Fig. 5:**
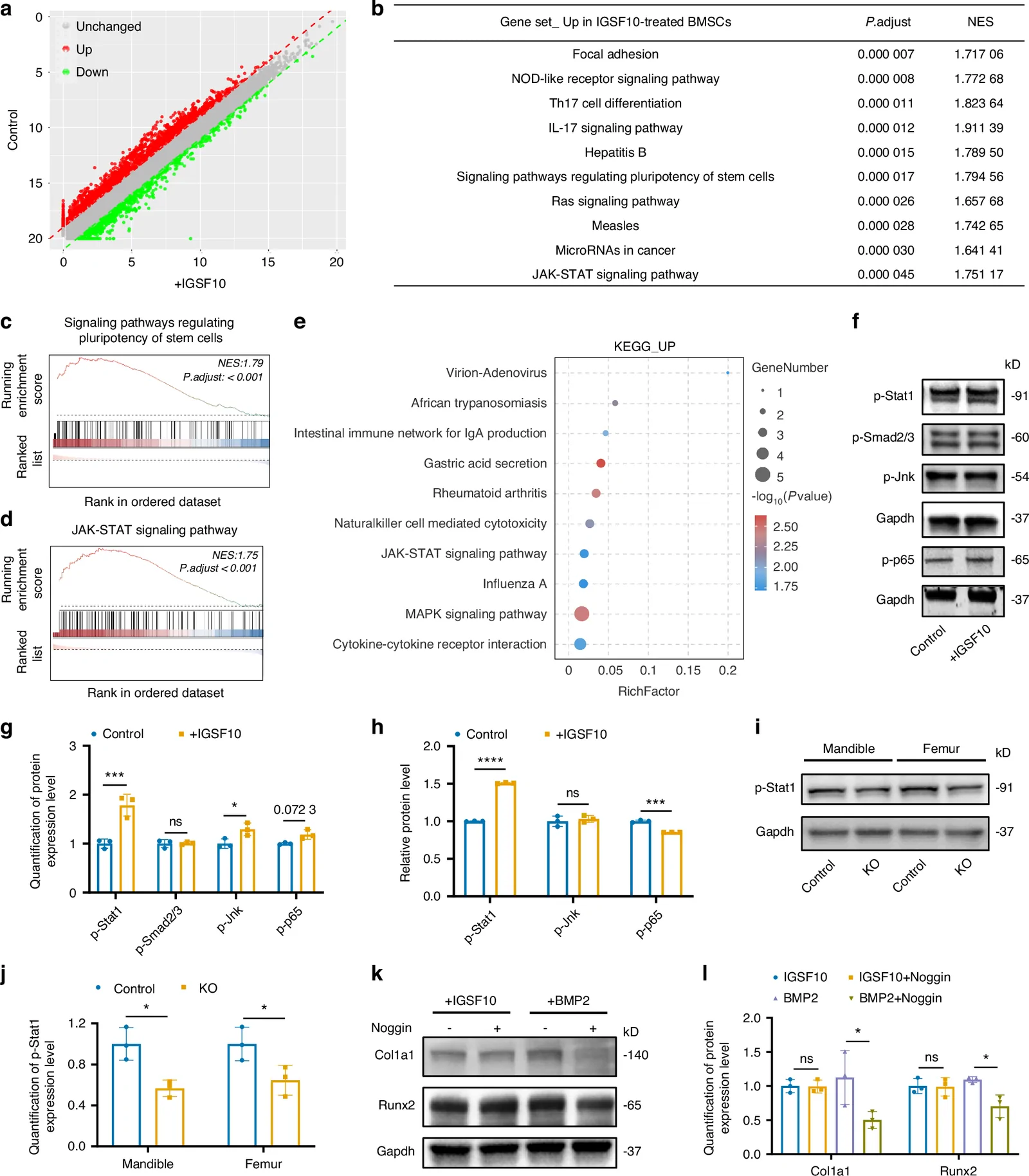
IGSF10 promotes osteogenesis via STAT1 activation independently of BMP2. **a** Volcano plot showing DEGs in BMSCs treated with IGSF10 for 3 days. **b** The top 10 upregulated gene sets in GSEA. GSEA plots for the “Signaling pathways regulating pluripotency of stem cells” (**c**) and “JAK-STAT pathways” (**d**) gene sets. **e** KEGG pathway enrichment analysis of upregulated DEGs. **f** Western blot analysis of p-Stat1, p-Smad2/3, p-Jnk, and p-p65 in BMSCs treated with IGSF10. **g** Quantification of protein phosphorylation levels in (**f**) (*n* = 3). **h** Quantification of protein levels by ELISA (*n* = 3). **i** Western blot analysis of p-Stat1 in mandibles and femurs from control and KO mice. **j** Quantification of p-Stat1 protein levels in (**i**) (*n* = 3). **k** Western blot analysis of Col1a1 and Runx2 in BMSCs. **l** Quantification of protein expression levels in (**k**) (*n* = 3)

To delineate the mechanism underlying IGSF10-driven osteogenesis, we performed RNA-seq in wild-type BMSCs treated with 100 ng/mL IGSF10 for 3 days. We identified 234 differentially expressed genes (fold change ≥2, *P* < 0.05) (Fig. 5a). GSEA revealed enrichment of gene sets involved in stem cell pluripotency and intracellular signaling (Fig. 5b–d). KEGG analysis highlighted JAK-STAT and MAPK-related pathways among the most significantly enriched, whereas canonical osteogenic pathways such as BMP, Wnt, and TGF-β were not enriched (Fig. 5e).

Based on these results, we examined pathway activation at the phospho-protein level. Western blotting showed robust Stat1 phosphorylation in response to IGSF10, while phosphorylation changes in Smad2/3, Jnk, and p65 were minimal and/or not consistently observed under the tested conditions (Fig. 5f, g). Enzyme-linked immunosorbent assay (ELISA) further confirmed Stat1 activation, showing a 1.51-fold increase in p-Stat1 concentration (*P* < 0.000 1; Fig. 5h). Consistently, bone tissues from KO mice exhibited reduced phospho-Stat1 abundance (Fig. 5i, j).

To test BMP pathway dependency, we compared IGSF10 and BMP2 responses in the presence of the BMP antagonist Noggin. As expected, Noggin abolished BMP2-induced Col1a1 and Runx2 upregulation, whereas IGSF10 retained osteoinductive activity under Noggin treatment (Fig. 5k, l).

### IGSF10 acts via EGFR, activating STAT1 in osteoblasts while suppressing osteoclasts

**Fig. 6:**
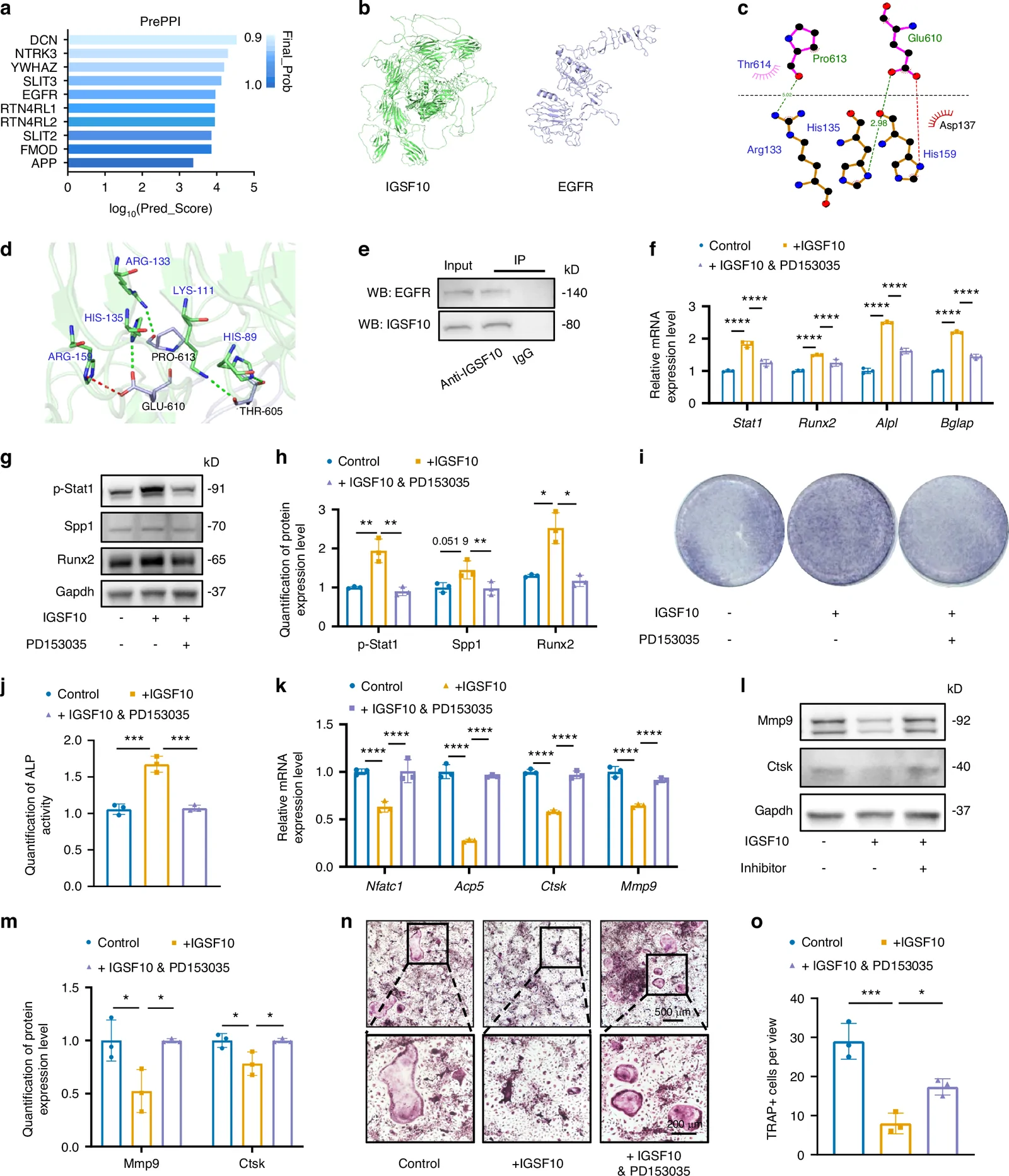
IGSF10 engages EGFR to regulate osteoblast and osteoclast function. **a** Predicted interaction proteins for IGSF10 from the PrePPI database. Molecular docking model of the IGSF10-EGFR complex: overall structure (**b**), detailed interaction interface at the core binding site (**c**), and schematic diagram of molecular interactions (**d**). **e** Co-IP assay confirming the interaction between IGSF10 and EGFR. **f** mRNA expression of *Runx2, Col1a1, Alpl* and *Stat1* in BMSCs by qRT-PCR (*n* = 3). **g** Western blot analysis of p-Stat1 and osteogenic marker proteins (Spp1, Runx2) in BMSCs. **h** Quantification of protein expression levels in (**g**) (*n* = 3). **i** ALP staining of BMSCs. **j** Quantification of ALP activity (*n* = 3). **k** mRNA expression of osteoclast markers (*Nfatc1, Acp5, Ctsk, Mmp9*) in BMMs by qRT-PCR (*n* = 3). **l** Western blot analysis of Mmp9 and Ctsk protein levels in BMMs. **m** Quantification of protein expression levels in (**l**) (*n* = 3). **n** TRAP staining of BMM-derived osteoclasts. **o** Quantification of TRAP-positive multinucleated osteoclasts per view (*n* = 3)

Because IGSF10 is secreted, we sought to identify its membrane receptor. PrePPI analysis predicted candidate interactors (Fig. 6a); EGFR was prioritized based on its functional relevance to phosphorylation signaling and development (Fig. S5) and its documented ability to engage STAT1.

Molecular docking supported a putative IGSF10–EGFR interaction, with both a favorable docking score (–294.07) and a high confidence score (0.947) indicating specific physical binding. The interaction between the two protein regions is stabilized by multiple hydrogen bonds, hydrophobic interactions, and aromatic contacts, with His135, Arg133, His159, and Lys111 forming the core binding interface (Fig. 6b–d, the full interaction network is provided in Fig. S6), and co-immunoprecipitation confirmed an association between IGSF10 and EGFR (Fig. 6e). Functionally, EGFR inhibition with PD153035 abrogated IGSF10-induced upregulation of osteogenic genes and *Stat1* (Fig. 6f), reduced p-Stat1 and osteogenic marker proteins (Spp1, Runx2) (Fig. 6g, h), and diminished ALP activity (Fig. 6i, j).

In osteoclast cultures, IGSF10 decreased expression of osteoclast markers (Fig. 6k–m) and reduced TRAP-positive multinucleated cell formation (Fig. 6n, o). In bone slice assays, IGSF10 reduced the total resorption area by 66.41% (*P* < 0.000 1; Fig. S7b), and SEM revealed smaller, more discrete pits rather than extensive trench-like resorption (Fig. S7a). These inhibitory effects were substantially reversed by PD153035, with the resorption area restored to 82.62% ± 7.83% of the control level. Notably, STAT1 activation was not detected in BMMs under these conditions, suggesting an EGFR-dependent but STAT1-independent downstream mechanism in the osteoclast lineage.

### Bone regeneration efficacy of IGSF10 and its synergy with BMP2

**Fig. 7:**
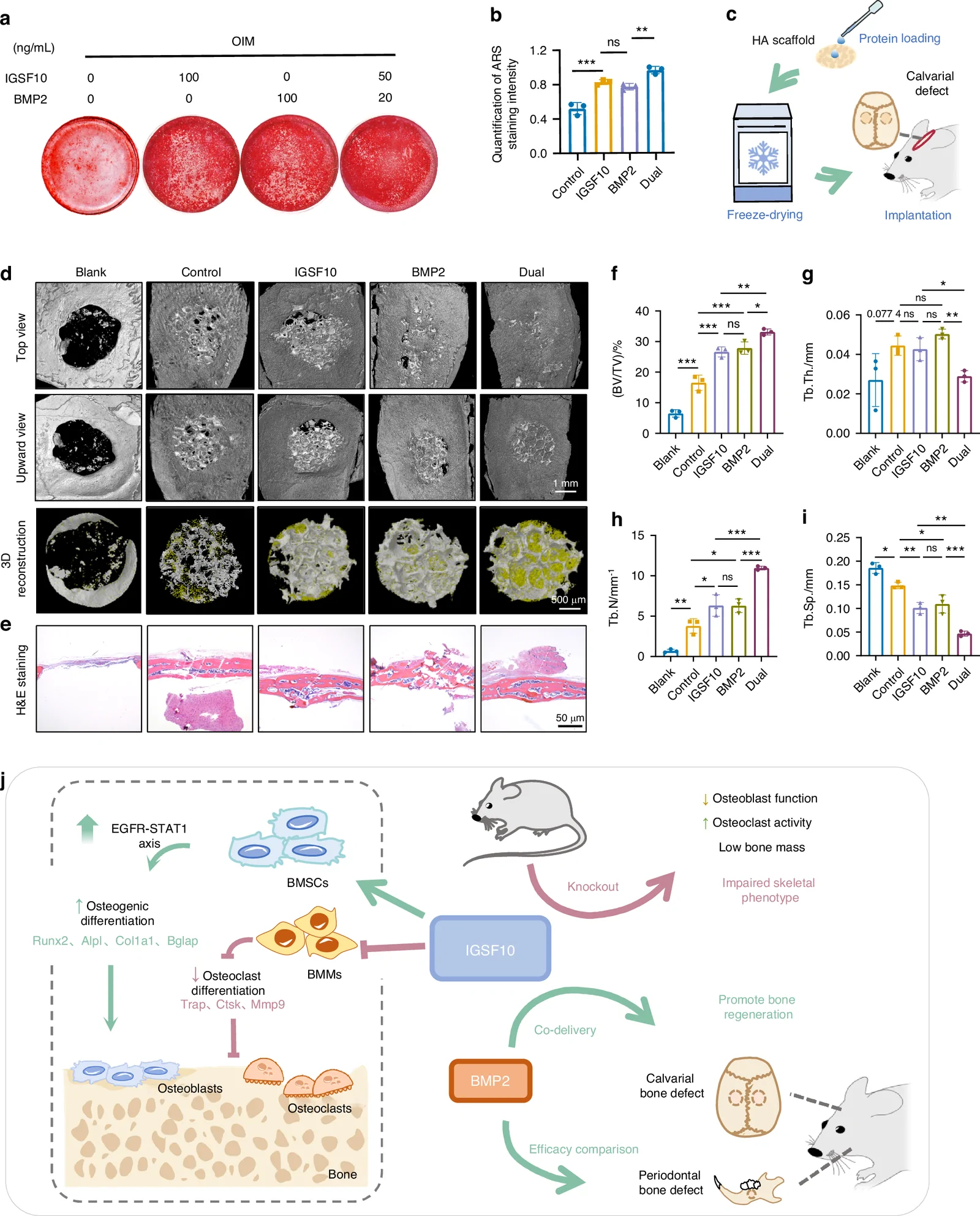
IGSF10 synergizes with BMP2 to enhance bone regeneration. **a** ARS staining of BMSCs treated with suboptimal concentrations of IGSF10 and/or BMP2 under OIM for 14 days. **b** Quantification of ARS staining (*n* = 3). **c** Schematic diagram illustrating the establishment of the mouse critical-sized calvarial defect model. **d** Representative 3D micro-CT reconstructions of calvarial defects at 8 weeks post-surgery (newly formed bone is highlighted in yellow). **e** H&E staining of calvaria. Quantitative micro-CT analysis of the regenerated bone within the defect region: BV/TV (**f**), Tb.Th (**g**), Tb.N (**h**), and Tb.Sp (**i**) (*n* = 3). **j** Graphic abstract illustrating the proposed mechanism of action for IGSF10 in bone homeostasis and regeneration

To evaluate regenerative potential, we tested the interaction between IGSF10 and BMP2. In vitro, under OIM, co-treatment with IGSF10 significantly enhanced mineral deposition induced by suboptimal BMP2 concentrations (Fig. 7a, b), consistent with a cooperative effect under osteogenic conditions.

We next assessed this interaction in a critical-sized calvarial defect model. Groups included blank (defect only), scaffold control, and scaffolds loaded with IGSF10, BMP2, or both (Fig. 7c). Micro-CT at 8 weeks demonstrated improved defect bridging in the dual-treatment group compared with single-factor treatments (Fig. 7d). In the micro-CT reconstructions, voxels segmented within the defined grayscale range for newly formed bone were color-coded in yellow (as described in Methods), facilitating separation of regenerated tissue from background.

Quantitative micro-CT analysis further supported enhanced regeneration with combined treatment (Fig. 7f–i). Compared with the scaffold control [BV/TV: (16.54 ± 2.509)%; Tb.N: 3.786 ± 0.909 mm⁻¹; Tb.Sp: 0.149 ± 0.007 mm], both IGSF10 alone [BV/TV: (26.63 ± 1.651)%; Tb.N: 6.319 ± 1.342 mm⁻¹; Tb.Sp: 0.101 ± 0.011 mm] and BMP2 alone [BV/TV: (27.87 ± 2.057)%; Tb.N: 6.294 ± 0.833 mm⁻¹; Tb.Sp: 0.109 ± 0.019 mm] significantly increased BV/TV and Tb.N while reducing Tb.Sp. Notably, the dual treatment achieved the highest BV/TV [(33.15 ± 1.007)%] and Tb.N (10.960 ± 0.231 mm⁻¹), alongside the lowest Tb.Sp (0.047 ± 0.005 mm), exceeding the levels attained by either single‑factor group. Tb.Th was not increased by the single treatments and was slightly lower in the dual group (0.029 ± 0.003 mm) compared with the scaffold control (0.044 ± 0.005 mm), consistent with the formation of a greater number of finer trabeculae during active remodeling within the regenerated volume. H&E staining corroborated the imaging findings and showed more extensive new bone formation in the dual group (Fig. 7e).

## Discussion

This study expands the functional landscape of IgSF proteins in the skeleton by identifying IGSF10 as an extracellular regulator that can shift the remodeling balance toward a more bone-anabolic state (Fig. 7j). A range of established pathways are known to affect both osteoblast and osteoclast programs (e.g., RANKL/OPG, sclerostin/Wnt, TGF-β, IGF-1, Semaphorin, and Eph/Ephrin signaling).^14,17,29,30^ However, many of these signals are broadly pleiotropic and embedded in complex feedback loops, which can complicate their therapeutic use and make spatial–temporal control difficult.^31^ In this context, our findings suggest that IGSF10 may act as a more “coupling-relevant” extracellular cue—promoting osteogenic progression while concurrently restraining osteoclastogenesis—and therefore offers a conceptual basis for regeneration strategies that aim to coordinate formation and resorption rather than enhancing one arm alone.

A notable feature of the in vivo phenotype is that IGSF10 effects are not identical across skeletal sites. Differences between craniofacial and appendicular compartments likely reflect known biological contrasts in embryonic origin, ossification mode, and local mechanical/inflammatory environments.^32–34^ Thus, the comparatively modest calvarial response in certain indices should not be interpreted as a lack of function, but rather as evidence that IGSF10 output is context dependent. Importantly, the persistence of trabecular alterations in a mature cohort supports a role beyond early postnatal modeling, which aligns better with therapeutic scenarios that aim to modulate remodeling in an established skeleton (Fig. S2). These observations motivate future work mapping IGSF10 activity across age, anatomical site, and loading conditions, including controlled mechanical paradigms given prior links between IGSF10 and mechanosensitive osteogenesis.^35^

In vitro, IGSF10 did not behave as a “switch” that forces osteogenic fate under basal conditions. Instead, it mainly amplified osteogenic programs under permissive induction cues, consistent with a model in which IGSF10 accelerates lineage progression once transcriptional priming is present (Fig. 3a–b). Time-course profiling supports this view, showing earlier and/or stronger induction of osteogenic markers during differentiation (Fig. 3f), without detectable impairment of viability or proliferation under the tested conditions (Fig. 3g). At the cellular-behavior level, increased spreading (Fig. 3j–k) together with reduced clonogenicity (Fig. 3l–m) is compatible with a shift from self-renewal toward differentiation.^36^ However, these phenotypes, while informative, remain associative. Establishing causality will require targeted perturbation of adhesion–tension pathways (e.g., integrin–FAK modules and cytoskeletal regulators^5,37,38^) and time-resolved mapping of downstream transcriptional programs.

The strong osteogenic responsiveness in PDLCs suggests that IGSF10 may be particularly relevant for periodontal regeneration (Fig. 4). PDLCs reside in a mechanically active periodontal niche and differ from mesoderm-derived BMSCs in developmental origin and signaling biases;^39,40^ these factors could shape the kinetics and magnitude of IGSF10 responses without implying lineage exclusivity. From a translational perspective, the periodontal defect model highlights two attractive features under local delivery: (i) robust defect fill and (ii) reduced mineralization beyond the defined defect boundary compared with BMP2 under the present conditions (Fig. 4t). While encouraging for anatomically constrained applications, broader evaluation across dosing regimens, delivery systems, sites, and longer follow-up windows will be needed to define durability and safety margins.

In addition to its osteoinductive activity, our data support a consistent anti-resorptive role of IGSF10. In osteoclast lineage cultures, IGSF10 reduced osteoclast differentiation and diminished resorptive activity, as evidenced by fewer TRAP-positive multinucleated osteoclasts, reduced CTSK-associated osteoclastic activation, and functional resorption assays on bone slices (toluidine blue staining and SEM imaging of resorption lacunae). Together, these results indicate that IGSF10 affects both osteoclast formation and function, reinforcing the conclusion that it can coordinately modulate both arms of bone remodeling.

Mechanistically, our data support an EGFR-linked signaling framework in osteogenic cells, with STAT1 as a prominent downstream effector (Figs. 5–6). Two aspects are worth emphasizing. First, functional uncoupling from canonical BMP signaling (Noggin resistance^41^) argues that IGSF10 is not simply acting through BMP–Smad signaling, providing a mechanistic rationale for combination strategies with BMP2 (Fig. 5k–l). Second, the signaling output appears biased rather than broadly activating multiple osteogenic cascades: under our tested conditions and time window, STAT1 phosphorylation was robust, whereas canonical MAPK/Smad activation was not consistently detected. This supports a preferential route in the examined context rather than absolute pathway exclusion (Fig. 5f–g).^42^

While the EGFR–STAT1 axis was defined primarily in osteogenic lineage cells,^43^ inhibitor-based experiments suggest that EGFR activity also contributes to IGSF10-mediated suppression of osteoclastogenesis and resorption. Notably, STAT1 activation was not detectably induced in BMMs under our conditions (Fig. 6k–o; Fig. S5), pointing to an EGFR-dependent but potentially STAT1-independent mechanism in this lineage. This divergence highlights an important future direction: to determine how an upstream EGFR-associated cue can generate distinct downstream outputs in osteoblast versus osteoclast lineages, given the different core signaling architectures that govern osteoclastogenesis (e.g., M-CSF/c-Fms and RANKL/RANK pathways^44,45^).

From a regenerative standpoint, an actionable implication is mechanistic complementarity with BMP2. BMP2 is clinically used but limited by dose-related adverse events, motivating approaches that preserve efficacy while lowering BMP burden.^10–12^ Because IGSF10 signals through a pathway distinct from canonical BMP–Smad signaling, combining IGSF10 with BMP2 is best viewed as a dose-sparing, synergy-seeking strategy rather than redundant co-delivery (Fig. 7a–i). The critical-sized calvarial defect model was chosen as an intramembranous, low-load system to reduce biomechanical variability and thus provide a rational and controlled baseline for assessing intrinsic osteoinductive capacity independent of mechanical loading, while establishing a baseline for future studies incorporating controlled loading paradigms.

Several limitations should be acknowledged. (i) Although cohort sizes were increased for key systemic phenotyping, animal numbers in some defect experiments remained constrained; we therefore rely on concordant evidence across independent readouts and avoid over-interpreting modest effects. (ii) Mechanistic depth is greater in osteogenic cells than in the osteoclast lineage; defining EGFR-dependent downstream mediators in osteoclasts is an important next step. (iii) Docking and co-IP support an association between IGSF10 and EGFR but do not define binding kinetics or topology; biophysical assays and structural mapping will be required. (iv) Candidates emerging from our protein-level in silico prediction analyses that are linked to matrix remodeling programs (e.g., decorin) may contribute to downstream regenerative outputs, but causal validation will require targeted perturbation studies.

## Conclusion

In summary, this work supports IGSF10 as an extracellular osteoregulatory factor that promotes osteogenic progression while restraining osteoclastogenesis, acting through an EGFR-linked, STAT1-biased axis in osteogenic cells and exhibiting functional complementarity with BMP2 in regeneration models (Fig. 7j). These findings provide a foundation for future efforts to define the IGSF10–EGFR interaction mode, resolve osteoclast-lineage signaling, optimize delivery and dosing, and evaluate dose-sparing combination regimens for craniofacial and skeletal repair.

## Materials and methods

### Experimental design

This study aimed to investigate the role of IGSF10 in bone homeostasis and regeneration. Both loss-of-function and gain-of-function approaches were employed. *Igsf10* knockout mice were used to evaluate systemic bone phenotypes. Gain-of-function effects were tested using recombinant IGSF10 protein in both in vitro cultures (BMSCs, BMMs, PDLCs, THP-1) and in vivo bone defect models (periodontal and calvarial). Mechanistic studies explored EGFR–STAT1 signaling involvement.

All animal experiments were conducted in accordance with the “National Institutes of Health (NIH) Guide for the Care and Use of Laboratory Animals” and approved by the Institutional Animal Care and Use Committee at Ninth People’s Hospital, School of Medicine, Shanghai Jiao Tong University (Approval number: SH9H-2025-A1569-1). Human PDLCs-related experiments ethics approval for this study was granted by the Ethics Committee of the Ninth People’s Hospital affiliated with the Shanghai Jiao Tong University School of Medicine (Approval number: SH9H-2022-T393-1).

### Animal models

Mice and rats were housed under specific pathogen-free (SPF) conditions in a controlled environment [temperature: (22 ± 1) °C, humidity: (50 ± 10)%, 12-h light/dark cycle] with ad libitum access to standard chow and water. Both male and female animals were included. As the study was not powered to assess sex-specific differences, data were pooled across sexes for all analyses. All animals were acclimatized for at least one week prior to any experimental procedures.

#### *Igsf10*-knockout mouse model

C57BL/6JCya-*Igsf10*^em1flox^/Cya mice and C57BL/6JCya-TG(CAG-CreERTM)/Cya mice from GemPharmatech (Nanjing, China) were used and cross-bred in this study. Genotyping was performed by PCR on tail-tip DNA to confirm the presence of both *Igsf10*-floxed alleles and the CAG-CreERT2 transgene. At 2 weeks of age, offspring carrying both *Igsf10*-floxed alleles and CAG-CreERT2 were intraperitoneally administered tamoxifen (50 mg/kg body weight, dissolved in corn oil; Selleck, USA, S1238) once daily for 5 consecutive days to induce Cre-mediated recombination. Littermates carrying only the *Igsf10*-floxed alleles but not CAG-CreERT2 served as controls. Mice were euthanized at 4 weeks of age, and tissues were harvested for subsequent molecular and phenotypic analyses (*n* ≥ 5 each group).

#### Periodontal defect rat model

A total of 9 adult Sprague-Dawley rats (12 weeks old, GemPharmatech, China) were used. Animals were randomly assigned to three groups (*n* = 3 each): (i) Control (collagen scaffold alone), (ii) IGSF10 (scaffold loaded with IGSF10), and (iii) BMP2 (scaffold loaded with BMP2). A critical-sized periodontal defect (3 × 2 × 1 mm) was surgically created at the distal root of the mandibular first molar using a round bur (1.5 mm diameter) under general anesthesia induced by intraperitoneal injection of Zoletil 50 (40 mg/kg) and Domitor (0.25 mg/kg) and local analgesia (lidocaine infiltration). Collagen gel scaffolds loaded with recombinant IGSF10 (Abcam, USA, ab166199; 1 500 ng/mL, 100 µL) or BMP2 (R&D Systems, USA, 355-BM; 1 500 ng/mL, 100 µL) were implanted into the defect sites. The mandibles were harvested from each rat following euthanasia on day 21.

#### Calvarial defect mouse model

A total of 15 C57BL/6J mice (8 weeks old, GemPharmatech, China) were used. Mice were randomly assigned to five groups (*n* = 3 each): (i) Blank, (ii) Control (Hydroxyapatite (HA) scaffold), (iii) IGSF10 (HA + IGSF10), (iv) BMP2 (HA + BMP2), and (v) Dual (HA + IGSF10 + BMP2). After anesthesia (via intraperitoneal injection of Zoletil 50, 40 mg/kg, and Domitor, 0.5 mg/kg), a circular full-thickness calvarial defect (4 mm diameter) was created bilaterally using a trephine drill under constant saline irrigation. Macroporous HA scaffolds were fabricated via slurry casting and particle leaching. Briefly, a slurry of hydroxyapatite powder (Merck Millipore, 677418) was cast into a template filled with sucrose porogen (200–300 µm), followed by drying, porogen leaching in water, and sintering at 1 100 °C. The sintered scaffolds were sterilized and then incubated with recombinant IGSF10 (400 ng/mL), BMP2 (400 ng/mL), or both in PBS. The protein-loaded scaffolds were then frozen and lyophilized in a vacuum freeze-dryer for 24 h prior to implantation to ensure stable protein incorporation and handling. At 8 weeks post-surgery, mice were euthanized, and calvarial specimens were harvested.

### Cell culture

#### Isolation and culture of mouse bone marrow-derived cells

Bone marrow cells were aseptically isolated from the femurs of 4-week-old C57BL/6J mice (GemPharmatech, China) or age-matched *Igsf10*-knockout (KO) mice and their littermate controls. Cells were suspended in α-minimum essential medium (α-MEM; Thermo Fisher Scientific, USA, 12571063) supplemented with 10% fetal bovine serum (FBS; Thermo Fisher Scientific, USA, A5670701) and 1% penicillin-streptomycin (Thermo Fisher Scientific, USA, 15140122). For BMSCs culture, non-adherent cells were removed after 48 h, and the medium was refreshed every 3 days until 80% confluency. BMSCs at passage 2–3 were used for all experiments. For BMMs isolation, non-adherent cells were collected after 24 h of seeding and cultured in the presence of 50 ng/mL macrophage colony-stimulating factor (M-CSF; Merck Millipore, SRP3110) for 3 days to enrich osteoclast precursors.

##### Culture of human cells

Primary human PDLCs were obtained from healthy extracted third molars (with informed consent and institutional review board approval) and cultured in Dulbecco’s Modified Eagle Medium (DMEM; Sunncell, SNM-002B) supplemented with 10% FBS and 1% penicillin-streptomycin. The THP-1 human monocytic cell line (Sunncell, SNL-044) was maintained in RPMI-1640 medium (Thermo Fisher Scientific, USA, 12633020) containing 10% FBS and 1% penicillin-streptomycin. All cells were maintained at 37 °C in a humidified atmosphere of 5% CO₂ and used within passages 3-5 (PDLCs) or under 10 (THP-1).

##### Osteogenic and osteoclastic differentiation

For osteogenic differentiation, BMSCs or PDLCs were seeded at an appropriate density. Upon reaching confluence, the growth medium was replaced with osteogenic induction medium (OIM), which consisted of the respective growth medium supplemented with 10 mmol/L β-glycerophosphate (Merck Millipore, G9422), 50 µg/mL ascorbic acid (Merck Millipore, A4403), and 100 nmol/L dexamethasone (Merck Millipore, D1756). The medium was replaced every 2–3 days. Cells were harvested at the indicated time points for RNA (day 3), protein (day 7), alkaline phosphatase (ALP) staining (day 7), or Alizarin Red S (ARS) staining (day 14), as described in the Results.

For osteoclastic differentiation, BMMs or THP-1 cells were seeded and stimulated with 50 ng/mL recombinant receptor activator of nuclear factor-κB ligand (RANKL, Merck Millipore, T3573) in combination with 50 ng/mL M-CSF. The differentiation medium was refreshed every 2 days. Cells were harvested for RNA and protein analysis after 7 days, and for TRAP staining and bone resorption assays on bone slices after 14 days of induction, as detailed in the Results.

##### Treatment with recombinant protein and inhibitors

To investigate the osteogenic effect of IGSF10, cells were treated with recombinant IGSF10 protein at a concentration of 100 ng/mL in OIM. To assess the role of the EGFR–STAT1 axis, the selective EGFR inhibitor PD153035 (Selleck, S6546) was added to the culture at a final concentration of 2 μmol/L simultaneously with IGSF10. Cells were harvested at the specified time points for downstream analysis.

### Micro-CT analysis

Mandibles, femurs, and calvaria obtained from experimental animals were scanned using a micro-CT system (Bruker, Belgium; SkyScan 1276) with the following parameters: 50 kV voltage, 450 µA current, and 9 µm isotropic voxel resolution. Raw projection images were reconstructed using NRecon software (Bruker, Belgium; v1.7.1.0). For mandibular analysis, a region of interest (ROI) spanning the alveolar bone adjacent to the first molar (1.0 × 1.0 × 0.5 mm^3^) was selected. For femoral metaphyseal trabecular bone, an ROI located 1.0 mm distal to the growth plate (1.0 × 1.0 × 2.0 mm^3^) was analyzed. In the periodontal defect model, the mandibular first molar defect region was scanned, whereas in the calvarial defect model, the full-thickness cranial defect area was scanned. To distinguish newly formed bone, grayscale thresholding was applied with reference to the soft-tissue background and mature cortical bone within the same sample. Scaffold material was first segmented using a higher grayscale threshold (65–220) and excluded to avoid confounding from the hydroxyapatite scaffold. Newly formed bone was then segmented using a fixed grayscale range (50–65), empirically determined from representative samples as approximately 30%–50% higher than the soft-tissue background but lower than mature cortical bone. The same thresholds were applied uniformly across all samples, and voxels within the newly formed bone range were color-coded in yellow for visualization. Three-dimensional morphometric parameters, including BV/TV, Tb.N, Tb.Th, Tb.Sp, and cortical parameters (Ct.Th and Ct.Ar, as applicable), were quantified using CTAn software (Bruker, Belgium; v1.16).

### Histological analysis

Femurs, mandibles, and calvaria were dissected, fixed in 4% paraformaldehyde, and decalcified in 10% EDTA (pH 7.4) for 3–4 weeks. Decalcified tissues were dehydrated through a graded ethanol series, cleared in xylene, and embedded in paraffin. Sections (4 μm thickness) were cut using a microtome (Leica, Germany, RM2245). Histological staining, including H&E (Beyotime, China, C0105S), Movat pentachrome (Abcam, USA, ab245884), Masson’s trichrome (Beyotime, China, C0189S), and TRAP (Servicebio, China, G1050), was performed according to manufacturers’ protocols. Stained sections were imaged with a microscope (ZEISS, Germany, Axio Scope A1) equipped with DP2-TWAIN imaging software (v3.0.0.6212) for subsequent quantitative histological analysis.

### Immunohistochemical staining

Tissue sections were sequentially rehydrated through xylene and graded ethanol solutions. Antigen retrieval was performed by heating sections in 0.05% trypsin solution at 37 °C for 30 min, followed by endogenous peroxidase inactivation with 3% hydrogen peroxide. Sections were incubated with primary antibodies against Osteocalcin (Ocn; Abcam, USA, ab133612, 1:200) or Cathepsin K (Ctsk; Abcam, ab19027; dilution 1:200) at 4 °C overnight, then treated with goat anti-rabbit IgG (H + L) secondary antibody (Beyotime, China, A0208, 1:500). Diaminobenzidine (DAB) chromogenic detection was conducted using a commercial kit (Beyotime, China, P0202) according to the manufacturer’s instructions. Counterstaining with hematoxylin was performed before dehydrating sections through ethanol and xylene gradients. Images were acquired using a Zeiss Axio Scope A1 microscope equipped with DP2-TWAIN imaging software (v3.0.0.6212).

### RNA isolation and qRT-PCR

Total RNA was isolated using TRIzol reagent (Thermo Fisher Scientific, USA, 15596018CN) following the manufacturer’s protocol. Complementary DNA synthesis was performed with the PrimeScript RT Master Mix (Takara, Japan, RR036A) under controlled thermal cycling conditions. Amplification reactions were carried out in a Roche LightCycler 480 system (v1.5.1.74) employing PrimeScript RT Reagent Kit (Takara, Japan, RR037A) for quantitative detection of target transcripts. GAPDH served as the endogenous reference gene for data normalization. Primer sequences are detailed in Table S1.

### ALP staining and activity assay

Cells were fixed in 4% paraformaldehyde at room temperature for 30 min, and washed thrice with PBS. For chromogenic detection, a working BCIP/NBT solution was freshly prepared following the manufacturer’s guidelines of the Alkaline Phosphatase (ALP) staining kit (Beyotime, China, C3206). The samples were then completely immersed in the staining solution and incubated for 30 min at room temperature in the dark. After PBS washes, stained cells were observed under the microscope, and scanned and photographed. For quantitative analysis, parallel cultures were lysed, and ALP activity in the supernatant was measured using the p-nitrophenyl phosphate (pNPP) substrate from the same kit (Beyotime, China, P0321S), following the manufacturer’s instructions.

### Alizarin red staining

Cells were fixed with 4% paraformaldehyde at room temperature for 30 min, and subsequently rinsed three times with PBS. A 40 mmol/L Alizarin Red S (ARS; Solarbio, China, G3283) solution was applied to each well and incubated for 15 min at room temperature with gentle agitation. After triple washes with double-distilled water (ddH_2_O) to eliminate unbound dye, calcium nodules were imaged using a Microscope (Nikon, Japan, Eclipse Ti-U) with NIS-Elements software (v4.5000.1117.0). For quantification, the bound dye was dissolved in 10% (w/v) cetylpyridinium chloride (CPC) solution for 30 min. The absorbance of the resulting solution was measured at 560 nm.

### Calcium concentration assay

Total calcium concentration of PDLCs were quantified using a colorimetric microplate assay (Abcam, USA, ab102505). PDLCs were harvested and lysed by sonication in Calcium Assay Buffer, followed by centrifugation to collect the supernatant. A standard curve was prepared from a 5 mmol/L Calcium Standard, diluted serially in ddH_2_O, and loaded in duplicate onto a clear 96-well plate. Samples and standards were mixed with Chromogenic Reagent and Calcium Assay Buffer, incubated in the dark, and absorbance was measured at 575 nm using a microplate reader. Calcium concentrations were calculated based on the standard curve after blank subtraction.

### TRAP staining

For TRAP staining, BMMs or THP-1 cells were seeded in 24-well plates and induced to differentiate into osteoclasts as described. After 14 days of induction, cells were fixed with 4% paraformaldehyde for 10 min at room temperature. Fixed cells were incubated with a TRAP staining solution (Beyotime, China, P0332) according to the manufacturer’s instructions for 1 h at 37 °C in the dark. After washing, TRAP-positive multinucleated cells (containing three or more nuclei) were imaged under a light microscope (Nikon, Japan, Eclipse Ti-U) and quantified using ImageJ software.

### Bone resorption assay

The bone resorption assay was performed using BMMs. Cells were seeded onto bone slices (Boneslices, UK, 250) placed in 96-well plates and induced for osteoclast differentiation for 14 days. Cells were then removed by sonication in 10% sodium hypochlorite solution. The bone slices were washed, stained with 1% toluidine blue for 3 min to visualize resorption areas, and imaged under a light microscope (Nikon, Japan, Eclipse Ti-U). For scanning electron microscopy (SEM) analysis, additional slices were air-dried, sputter-coated with gold, and examined using an SEM (ZEISS, Germany, GeminiSEM 300). The total resorption area was quantified from toluidine blue-stained images using ImageJ software.

### Western blot analysis

Total proteins were extracted using RIPA buffer (Beyotime, China, P0013B) supplemented with protease inhibitor cocktail (Beyotime, China, P1008). Protein concentrations were determined by Pierce BCA Protein Assay Kit (Thermo Fisher Scientific, USA, A65453), and equal amounts of lysates were electrophoresed on 4%–12% SurePAGE Bis-Tris gels (Genscript, China, M00657) under denaturing conditions. Proteins were transferred to PVDF membranes (Thermo Fisher Scientific, USA, 88520) in Tris-Glycine buffer (1 h, 55 V), followed by blocking with 3% BSA PBS containing 0.1% Tween 20. Membranes were probed with primary antibodies diluted in blocking buffer (GAPDH [1:5 000; Abcam, USA, ab8245], COL1A1 [1:1 000; Abcam, USA, ab138492], SPP1 [1:1 000; Abcam, USA, ab8448], RUNX2 [1:1 000; Thermo Fisher Scientific, USA, MA5-41185], IGSF10 [1:1 000; Abcam, USA, ab197671], MMP9 [1:1 000; Abcam, USA, ab283575], CTSK [1:1 000; Abcam, USA, ab187647], p-Stat1 [1:1 000; Cell Signaling Technology, USA, 9167], p-Smad2/Smad3 [1:1 000; Cell Signaling Technology, USA, 8828], p-Jnk [1:1 000; Cell Signaling Technology, USA, 4668], p-p65 [1:1 000; Cell Signaling Technology, USA, 3033]) overnight at 4 °C. After washing, membranes were incubated with HRP-conjugated secondary antibodies (goat anti-mouse IgG (H + L) [1:5 000; Beyotime, China, A0216], goat anti-rabbit IgG (H + L) [1:5 000; Beyotime, China, A0208]) for 1 h at room temperature. Signals were visualized using ECL substrate (Proteintech, China, PK10003) and detected with UVITEC Alliance system (v16.0.3.0).

### CCK-8 assay

Cells were seeded in 96-well plates at a density of 1 × 10^3^ cells per well. At the designated time points, 10 µL of CCK-8 solution (Beyotime, China, C0037) was added to each well, followed by incubation for 1 h at 37 °C. The absorbance values were measured at 450 nm.

### Annexin/PI staining

After trypsinization, cells were resuspended in ice-cold PBS and stained with Annexin V-fluorescein isothiocyanate (FITC) and propidium iodide (PI) (Beyotime, China, C1062S) according to the manufacturer’s protocol. Stained cells were analyzed using a flow cytometer (Beckman Coulter, USA, CytoFLEX), and data were processed with Flowjo software (v10.8.1). Live cell populations were gated on forward scatter (FSC) versus side scatter (SSC) density plots to exclude debris and dead cells.

### Cell adhesion and spreading assessment

Cells were seeded at a density of 1 × 10^4^ cells per well. To evaluate cell adhesion and spreading, samples were fixed and stained at two time points: 2 h and 4 h. At each time point, cells were gently washed with PBS and fixed with 4% paraformaldehyde for 15 min at room temperature. After fixation, cells were permeabilized using 0.1% Triton X-100 in PBS for 5 min. Then samples were blocked with 1% BSA in PBS for 30 min to prevent non-specific binding. For cytoskeletal staining, cells were incubated with Alexa Fluor 488-conjugated phalloidin (1:200 dilution in PBS; Yeasen, China, 40736ES75) for 30 min at room temperature. Nuclear staining was performed using 4’,6-diamidino-2-phenylindole (DAPI; 1 µg/mL in PBS; Thermo Fisher Scientific, USA, 62248) for 5 min. After staining, samples were washed with PBS. Fluorescence images were captured using a fluorescence microscope (Nikon, Japan, Eclipse Ti-U).

### Colony-forming unit assay

Cells were trypsinized, adjusted to a density of 1 × 10^4^ cells/mL, and plated in culture dishes containing complete medium to ensure uniform distribution. After 7 days of incubation under standard conditions (37 °C, 5% CO_2_), macroscopically visible colonies were gently rinsed with PBS, fixed with 4% paraformaldehyde for 30 min at room temperature, and stained with 0.1% crystal violet (Merck Millipore, Germany, v5265) for 20 min. Excess dye was removed by thorough PBS rinsing. Colonies containing ≥ 50 cells were counted and documented.

### ELISA

The levels of p-STAT1, p-JNK, and p-p65 in cell lysates were quantified using commercial ELISA kits (p-STAT1: Cell Signaling Technology, USA, 73582; p-JNK: Cell Signaling Technology, USA, 7325; p-p65: Cell Signaling Technology, USA, 7173) according to the manufacturers’ instructions. Standard solutions, wash buffer, detection antibody working solution, and HRP-conjugated streptavidin working solution were prepared strictly following the manufacturer’s protocol. Standards and samples (100 μL/well) were loaded into pre-designated wells, mixed gently, and incubated at 37 °C for 2 h. After washing, 100 μL of HRP-streptavidin solution was added to each well, and the plate was sealed and incubated at 37 °C for an additional 1 h. Following substrate addition (90 μL/well), the plate was protected from light and incubated at 37 °C for 15–30 min for chromogenic development. Reactions were terminated with 50 μL stop solution, and optical density (OD) at 450 nm was measured within 5 min using a microplate reader (Tecan, Switzerland, Spark).

### RNA-sequencing (RNA-seq) analysis

Total RNA was extracted from treated BMSCs and sequenced on the Illumina NovaSeq 6000 platform. Raw reads were aligned to the reference genome, and gene-level read counts were quantified using HTSeq (v0.9.1), followed by normalization to fragments per kilobase of transcript per million mapped reads (FPKM). Differentially expressed genes (DEGs) were identified with DESeq (v1.30.0) using thresholds of |fold change | ≥2 and *P* < 0.05. KEGG pathway enrichment analysis of DEGs was performed using ClusterProfiler (v3.4.4, *P* < 0.05). Gene set enrichment analysis (GSEA, v4.1.0, Linux) was applied to annotate functional pathways and evaluate statistically enriched signaling cascades.

### Molecular docking analysis

The full-length 3D structures of IGSF10 and EGFR were predicted from their amino acid sequences using AlphaFold2. Protein-protein docking was performed using the HDOCK server to explore potential binding regions and interaction patterns. The top-ranked complex based on ITScorePP scoring was selected for further analysis. Structural visualization and interface analysis were conducted using PyMOL.

### Co-immunoprecipitation (Co-IP)

Cells were transfected with plasmids encoding full-length IGSF10 using Lipofectamine™ 3000 Transfection Reagent Kit (Thermo Fisher Scientific, USA, L3000008) according to the manufacturer’s instructions. After 48 h, cells were lysed in NP-40 Lysis Buffer (Beyotime, China, P0013F) containing protease inhibitors. The lysates were incubated overnight at 4 °C with anti-IGSF10 antibody or normal mouse IgG (Santa Cruz Biotechnology, USA, SC-2025) as a negative control, followed by incubation with Protein A/G magnetic beads (Thermo Fisher Scientific, USA, 88802) overnight at 4 °C. After extensive washing, immunoprecipitated proteins were eluted and subjected to Western blot analysis using anti-EGFR antibody (Santa Cruz Biotechnology, USA, SC-120) to detect protein–protein interactions.

### Statistical analysis

During outcome assessment and statistical analysis, evaluators were blinded to group allocation. All experiments were repeated three or more times independently, and statistical analyses were performed using GraphPad Prism (v8.0.2). For comparisons between two groups, a two-tailed unpaired Student’s t-test was used. For comparisons among multiple groups, one-way ANOVA followed by Tukey’s post hoc test was applied; where data did not meet parametric assumptions, the Kruskal–Wallis test with Dunn’s multiple-comparisons correction was used. Data are presented as mean ± SD. Significance levels were indicated as follows: *P* value < 0.05 (*), *P* value < 0.01 (**), *P* value < 0.001 (***), and *P* value < 0.000 1 (****); ns, not significant. The number of independent experiments (n) is specified in figure legends.

## Supplementary information

Supplementary figure 1

Supplementary figure 2

Supplementary figure 3

Supplementary figure 4

Supplementary figure 5

Supplementary figure 6

Supplementary figure 7

Supplementary table 1

Supplementary figure Legend

## Data Availability

All data are presented in this manuscript. The sequencing data generated in this study are available in the NCBI database under the accession number PRJNA1294145. Data that are mentioned but not shown in this manuscript are available from the corresponding author upon reasonable request.
