# Supplementary material for: IGSF10 regulates osteogenesis and osteoclastogenesis via a noncanonical EGFR–STAT1 signaling axis to promote skeletal regeneration
Source: Bone Res. 2026 Aug 4;14:81. doi: 10.1038/s41413-026-00552-2 (PMC13438563; doi:10.1038/s41413-026-00552-2)
Supplement: Supplementary file 1 [file 41413_2026_552_MOESM8_ESM.docx]

Supplementary Table 1. Sequences of the primers used for qRT-PCR

| Genes | Forward primer | Reverse primer |
| --- | --- | --- |
| *Igsf10* | CTGAAGAGTCAGTCCCCCAG | CTCTCTTCTGCATCCTGAGC |
| *Sp7* | TGGGAACAAGAGTGAGCTGG | AGTGAGCTTCTTCCTCAAGCA |
| *Bglap* | TTGGCCCAGACCTAGCAGAC | CTGGGCTTGGCATCTGTGAG |
| *Col1a1* | CGATGGATTCCCGTTCGAGT | CGATCTCGTTGGATCCCTGG |
| *Runx2* | CCTCAGTGATTTAGGGCGCA | GCCTGCCTGGGATCTGTAAT |
| *Alpl* | CTGGACTTGGTGGTCACAGC | AATTGACGTTCCGATCCTGAGT |
| *Ctsk* | CACCCTTAGTCTTCCGCTCA | CTTGAACACCCACATCCTGC |
| *Mmp9* | CAGCCGACTTTTGTGGTCTTC | CGGTACAAGTATGCCTCTGCCA |
| *Tnfrsf11a* | GCATCCCTTGCAGCTCAACA | ATGGAAGAGCTGCAGACCAC |
| *Acp5* | GCCCTCTGCAACTCTGGACT | CCCATGAATCCATCTTGGCGG |
| *Nfatc1* | TCAGAGTGAGACCGAGAGGC | TGACATGCGGGGTGTGTG |
| *Stat1* | GCTCGTTTGTGGTGGAAAGAC | TCTCTCATTCACATCTCTCAACTT |
| *Gapdh* | ACCCTTAAGAGGGATGCTGC | CCCAATACGGCCAAATCCGT |
| *COL1A1* | TGAAGGGACACAGAGGTTTCAG | GTAGCACCATCATTTCCACGA |
| *BGLAP* | CACCGAGACACCATGAGAGC | CTGCTTGGACACAAAGGCTGC |
| *RUNX2* | CGCCTCACAAACAACCACAG | ACTGCTTGCAGCCTTAAATGAC |
| *ALPL* | CTATCCTGGCTCCGTGCTCC | TTAACTGATGTTCCAATCCTGCG |
| *NFATC1* | CACCGCATCACAGGGAAGAC | GCACAGTCAATGACGGCTC |
| *MMP9* | GGGACGCAGACATCGTCAT | TCGTCATCGTCGAAATGGGC |
| *CTSK* | GAGGCTTCTCTTGGTGTCCATAC | TTACTGCGGGAATGAGACAGGG |
| *GAPDH* | CAAGGCTGTGGGCAAGGTCATC | GTGTCGCTGTTGAAGTCAGAGGAG |
