# Supplementary material for: IGSF10 regulates osteogenesis and osteoclastogenesis via a noncanonical EGFR–STAT1 signaling axis to promote skeletal regeneration
Source: Bone Res. 2026 Aug 4;14:81. doi: 10.1038/s41413-026-00552-2 (PMC13438563; doi:10.1038/s41413-026-00552-2)
Supplement: Supplementary file 2 [file 41413_2026_552_MOESM9_ESM.docx]

**Supplementary Fig. 1 Analysis of femoral cortical bone at 4 weeks in KO mice.**

(a–b) Micro-CT quantification of cortical bone parameters: (a) cortical area (Ct.Ar) and (b) cortical thickness (Ct.Th) in the femurs of Control and KO mice (n=5).

**Supplementary Fig. 2 Analysis of femoral trabecular bone at 12 weeks in KO mice.**

(a–d) Micro-CT quantification of trabecular parameters: (a) BV/TV, (b) Tb.N, (c) Tb.Th, and (d) Tb.Sp in the femurs of Control and KO mice (n=5).

**Supplementary Fig. 3 Analysis of apoptosis in IGSF10-treated BMSCs.**

(a) Flow cytometry analysis of apoptosis via Annexin V/PI staining. (b) Quantification of apoptotic cells (n=3).

**Supplementary Fig. 4 Quantitative analysis of calcium concentration in cell lysates after osteogenic induction.**

**Supplementary Fig. 5 GO enrichment analysis of biological processes for the predicted IGSF10 interactome.**

**Supplementary Fig. 6 Comprehensive mapping of the protein-protein interaction interface.**

(a) Two-dimensional map of residue interactions between IGSF10 and EGFR. (b) Three-dimensional view of the interface showing the spatial arrangement of key residues.

**Supplementary Fig. 7 Analysis of osteoclast-mediated bone resorption.**

(a) Toluidine blue staining (up) and SEM (down) images of bone slices. The white dashed lines demarcate the resorption areas. (b) Quantification of the percentage of re
